# Beneficial effects of octreotide in alcohol-induced neuropathic pain. Role of H_2_S, BDNF, TNF-α and Nrf2

**DOI:** 10.1590/ACB360408

**Published:** 2021-05-31

**Authors:** Rongqiang Jiang, Hongxia Wei

**Affiliations:** 1 MM. The No. 4 People’s Hospital of Hengshui – Department of Anesthesiology – Hengshui City, China.; 2 MM. Ninth Hospital of Xi’an – Intensive Care Unit – Shaanxi, China.

**Keywords:** Ethanol, Neuralgia, Hyperalgia, Octreotide, Rats

## Abstract

**Purpose:**

To explore the role and molecular mechanisms of neuroprotective effects of octreotide in alcohol-induced neuropathic pain.

**Methods:**

Male Wistar rats were employed and were administered a chronic ethanol diet containing 5% v/v alcohol for 28 days. The development of neuropathic pain was assessed using von Frey hair (mechanical allodynia), pinprick (mechanical hyperalgesia) and cold acetone drop tests (cold allodynia). The antinociceptive effects of octreotide (20 and 40 µg·kg^–1^) were assessed by its administration for 28 days in ethanol-treated rats. ANA-12 (0.25 and 0.50 mg·kg^–1^), brain-derived neurotrophic factor (BDNF) receptor blocker, was coadministered with octreotide. The sciatic nerve was isolated to assess the biochemical changes including hydrogen sulfide (H_2_S), cystathionine β synthase (CBS), cystathionine γ lyase (CSE), tumor necrosis factor-α (TNF-α), BDNF and nuclear factor erythroid 2-related factor 2 (Nrf2).

**Results:**

Octreotide significantly attenuated chronic ethanol-induced neuropathic pain and it also restored the levels of H_2_S, CBS, CSE, BDNF, Nrf2 and decreased TNF-α levels. ANA-12 abolished the effects of octreotide on pain, TNF-α, BDNF, Nrf2 without any significant effects on H_2_S, CBS, CSE.

**Conclusions:**

Octreotide may attenuate the behavioral manifestations of alcoholic neuropathic pain, which may be due to an increase in H_2_S, CBS, CSE, BDNF, Nrf2 and a decrease in neuroinflammation.

## Introduction

Alcohol is one of the most commonly abused substances in the world and the development of neuropathic pain is one of the most common serious complications of its chronic consumption^1^. Chronic alcohol consumption induces neuropathological changes^2^, which may have diverse manifestations, including the development of pain symptoms in the form of peripheral neuropathy^3^. However, there is no reliable pharmacological agent for its management and, thus, there is a need to explore new effective agents to ameliorate the symptoms of neuropathic pain.

Octreotide is a somatostatin analogue^4^ and it has been used clinically for the management of acromegaly, carcinoid syndrome, acute hemorrhage from esophageal varices in liver cirrhosis, acute pancreatitis, refractory hypoglycaemia^5^^,^ 6. Apart from these, it has been found to produce other diverse actions including a decrease in ischemia-reperfusion-induced injury to kidney, liver, brain and heart^7^^,^ 8. The role of somatostatin receptors, localized on the peripheral primary afferent terminals, in the development of pain sensitization has been reported^9^^,^ 10. It is also found to attenuate pain in formalin-induced pain model^11^^,^ 12 and diabetic neuropathy model^13^. However, its role and molecular mechanisms in alcoholic neuropathy are not explored yet.

Brain-derived neurotrophic factor is a member of the neurotrophin family of growth factors and its role in the development of peripheral neuropathic pain has been reported^14^. It is involved in neuronal survival and its levels are found to be decreased in alcohol-induced neurotoxicity^15^. Hydrogen sulfide (H_2_S) is a gaseous neurotransmitter and it is mainly synthesized by cystathionine β synthase (CBS), cystathionine γ lyase (CSE). It has been found that the exogenous administration of H_2_S ameliorates alcohol-induced deleterious effects including neurotoxicity^16^. Furthermore, the role of neuroinflammatory mediators including tumor necrosis factor-α (TNF-α)^17^ and transcriptional factor regulating the endogenous antioxidant system, i.e., nuclear factor erythroid 2-related factor 2 (Nrf2)^18^ in neuropathic pain has been defined. Based on these, the present study was designed to explore the beneficial effects of octreotide in alcohol-induced neuropathic pain with a particular emphasis on the role of H_2_S, brain-derived neurotrophic factor (BDNF), TNF-α and Nrf2.

## Methods

### Animals, drugs and chemicals

The experimental protocol was approved by the Animal Ethical Committee of No.4 People’s Hospital of Hengshui, Ethic No. HB2020-11(05). All experiments were conducted as per the ethical guidelines of the Animal Ethical Committee.

Male Wistar albino rats were employed for the current study and were kept in the animal house of People’s Hospital of Hengshui. The animals were provided with standard feed and water. The animals were exposed to 12 h of light and 12 h of the dark at 25 ± 2 °C and 55–60% relative humidity. The ELISA kits for the quantification of BDNF (ab213899), TNF-α (ab236712) and Nrf2 (ab207223) were procured from Abcam, USA. The ELISA kit for CSE (abx155408) was procured from Abbexa LLC, Houston, USA; while the fluorometric assay kit for CBS (K-998) was obtained from BioVision, Inc, California USA. Octreotide and ANA-12 were procured from Sigma-Aldrich, USA.

### Induction of alcohol-induced neuropathic pain

The rats were administered a chronic ethanol diet containing 5% v/v alcohol for 28 days. In this study, rats were administered the Lieber-DeCarli diet (most commonly employed for alcohol feeding to rodents) for initial five days for acclimatization to liquid tube feeding. Thereafter, ethanol Lieber-DeCarli diet containing 5% v/v ethanol was administered daily via oral feeding tube (100 mL·day^–1^·rat^–1^) for 28 days^19^^,^ 20.

### Behavioral tests

The acclimatization of animals to laboratory apparatus is essential to reduce the variations during actual behavioral experimentation. The animals were kept in each apparatus for 5 min for three days before the start of actual experimentation.

#### Von Frey hair test for mechanical allodynia

Neuropathic pain is characterized by the development of mechanical allodynia, i.e., animals exhibit pain in response to nonpainful mechanical stimuli. Accordingly, the von Frey hair test (BiosebLab, France) was conducted to assess mechanical allodynia in which response of animals to von Frey hair filaments of different bending forces (0.008 to 300g). In this test, von Frey hair filaments (of varying stiffness) were applied ten times in the ascending order of stiffness to the plantar region of the hind paw to induce paw withdrawal. The withdrawal threshold was noted in grams, which was equal to von Frey hair stiffness that evoked 50% paw withdrawal^21^.

#### Acetone spray test for cold allodynia

Another characteristic feature of neuropathic pain is the development of cold allodynia in response to a non-noxious cold stimulus (e.g., acetone). In this test, acetone (100 μL) was sprayed on the plantar surface of the hind paw to evoke a paw withdrawal response. The total time for which the animal kept its paw in the air (paw withdrawal duration), after withdrawal in response to acetone application was noted in seconds^22^.

#### Pinprick test for mechanical hyperalgesia

In this test, the development of mechanical hyperalgesia, i.e., excessive pain in response to mechanical pain stimuli, was assessed using a pinprick test. For conducting this test, a pointed pin was applied to the plantar surface of the hind limb. The total time for which the animal kept its paw in the air (paw withdrawal duration), after withdrawal, in response to pinprick was noted in seconds^23^.

### Biochemical tests

After conducting behavioral tests on the 28^th^ day, rats were sacrificed by an overdose of 4.5% isoflurane (gaseous anesthetic agent) to isolate the sciatic nerve (kept at –70 °C till processing for biochemical analysis), which was homogenized in phosphate buffer saline (PBS), pH 7.4. The nerve homogenate was centrifuged at 2500 g for 30 min to remove sediments and retain supernatants. The levels of different biochemicals were quantified in the supernatants of nerve homogenate. The levels of H_2_S were quantified using reverse-phase chromatography^24^, while the levels of CBS were quantified using a fluorometric assay kit. In this test, cysteine and homocysteine were added to the supernatant of nerve homogenate to generate H_2_S, which was allowed to react with the azide-functional group to yield fluorescence. The fluorescence was detected using an excitation wavelength of 368 nm and an emission wavelength of 460 nm^25^. The levels of CSE, BDNF, TNF-α and Nrf2 were quantified using commercially available ELISA kits. The protein levels in the nerve homogenate were measured using Folin–Lowry’s method.

### Experimental design

Six groups were used and each group comprised eight animals:

(i) Control: animals received alcohol free calorie-matched diet (maltose-dextrin) for 28 days.
(ii) Ethanol-fed diet: animals received 5% v/v ethanolic diet for 28 days.
(iii) Octreotide (20 µg·kg^–1^) in ethanolic-fed diet: ethanolic fed-animals received 20 µg·kg^–1^ of octreotide for 28 days.
(iv) Octreotide (40 µg·kg^–1^) in ethanolic-fed diet: ethanolic fed-animals received 40 µg·kg^–1^ of octreotide for 28 days.
(v) ANA-12 (0.25 mg·kg^–1^) and octreotide (40 µg·kg^–1^) in ethanolic-fed diet: 0.25 mg·kg^–1^ of ANA-12, BDNF receptor antagonist, was administered along with octreotide (40 µg·kg^–1^) in ethanolic fed-animals for 28 days.
(vi) ANA-12 (0.5 mg·kg^–1^) and octreotide (40 µg·kg^–1^) in ethanolic-fed diet: 0.5 mg·kg^–1^ of ANA-12, BDNF receptor antagonist, was administered along with octreotide (40 µg·kg^–1^) in ethanolic fed-animals for 28 days.

### Statistical analysis

The data were represented as mean ± standard deviation. The statistical analysis was done using one-way analysis of variance (ANOVA). Thereafter, Tukey’s multiple comparison test was used for *post hoc* analysis. The p-value < 0.05 was considered to be statistically significant.

## Results

## Development of neuropathic pain symptoms in ethanolic-fed diet

**Figure 1:**
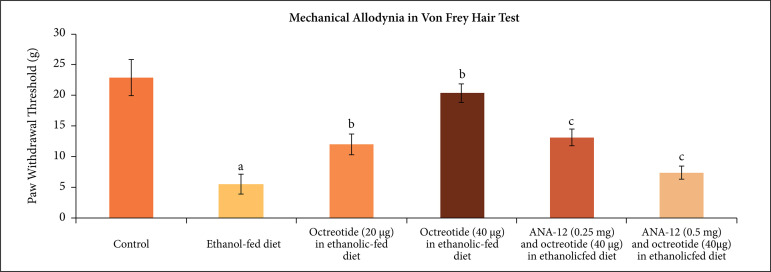
Effect of different treatments on mechanical allodynia as assessed by von Frey test. **a** = p < 0.05 *vs*. control; **b** = p < 0.05 *vs*. ethanol fed diet; **c** = p < 0.05 *vs*. octreotide (40 µg·kg^–1^) in ethanolic-fed diet.

**Figure 2:**
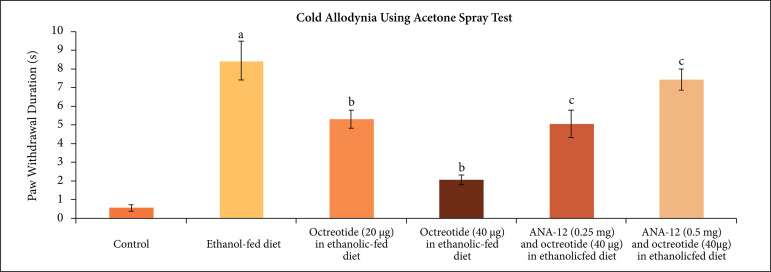
Effect of different treatments on cold allodynia as assessed by acetone spray test. **a** = p < 0.05 *vs*. control; **b** = p < 0.05 *vs*. ethanol fed diet; **c** = p < 0.05 *vs*. octreotide (40 µg·kg^–1^) in ethanolic-fed diet.

**Figure 3:**
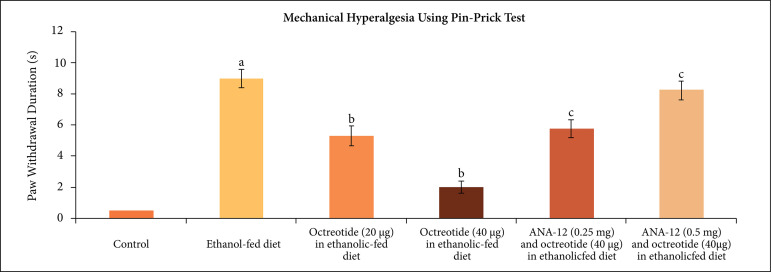
Effect of different treatments on mechanical hyperalgesia as assessed by pinprick test. **a** = p < 0.05 *vs*. control; **b** = p < 0.05 *vs*. ethanol fed diet; **c** = p < 0.05 *vs*. octreotide (40 µg·kg^–1^) in ethanolic-fed diet.

Administration of ethanolic diet (5% v/v) for 28 days led to a significant decrease in paw withdrawal threshold in von Frey hair test, suggesting the development of mechanical allodynia (Fig. 1), increase in paw withdrawal duration in acetone spray test, suggesting the development of cold allodynia (Fig. 2), increase in paw withdrawal duration in the pinprick test, suggesting the development of mechanical hyperalgesia (Fig. 3).

## Ethanolic-diet-induced neuropathic pain was associated with biochemical changes

**Figure 4:**
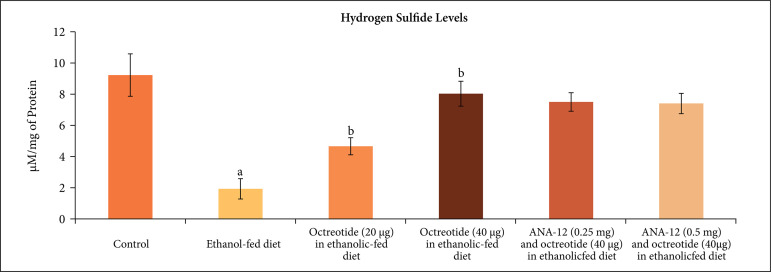
Effect of different treatments on hydrogen sulfide levels in the supernatant of nerve homogenate. **a** = p < 0.05 *vs*. control; **b** = p < 0.05 *vs*. ethanol fed diet; **c** = p < 0.05 *vs*. octreotide (40 µg·kg^–1^) in ethanolic-fed diet.

**Figure 5:**
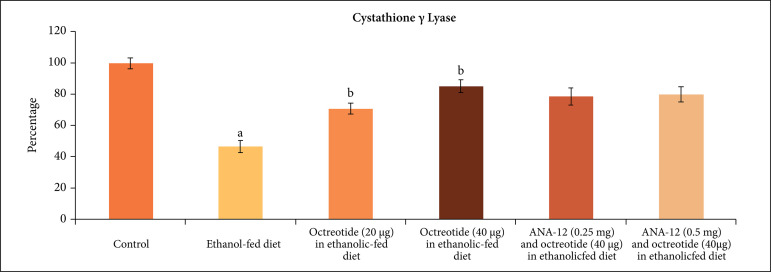
Effect of different treatments on cystathionine γ lyase levels in the supernatant of nerve homogenate. **a** = p < 0.05 *vs*. control; **b** = p < 0.05 *vs*. ethanol fed diet; **c** = p < 0.05 *vs*. octreotide (40 µg·kg^–1^) in ethanolic-fed diet.

**Figure 6:**
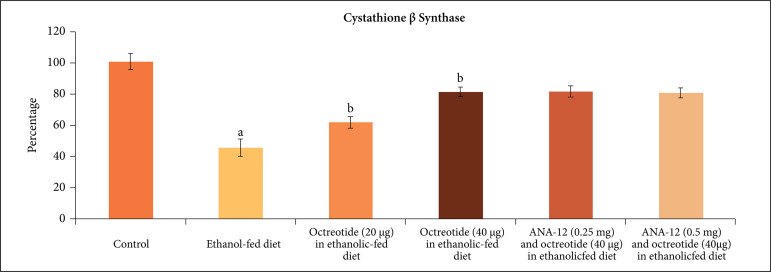
Effect of different treatments on cystathionine β synthase levels in the supernatant of nerve homogenate. **a** = p < 0.05 *vs*. control; **b** = p < 0.05 *vs*. ethanol fed diet; **c** = p < 0.05 *vs*. octreotide (40 µg·kg^–1^) in ethanolic-fed diet.

**Figure 7:**
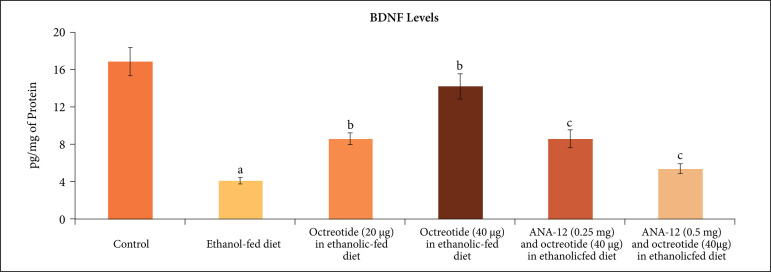
Effect of different treatments on the BDNF levels in the supernatant of nerve homogenate. **a** = p < 0.05 *vs*. control; **b** = p < 0.05 *vs*. ethanol fed diet; **c** = p < 0.05 *vs*. octreotide (40 µg·kg^–1^) in ethanolic-fed diet.

**Figure 8:**
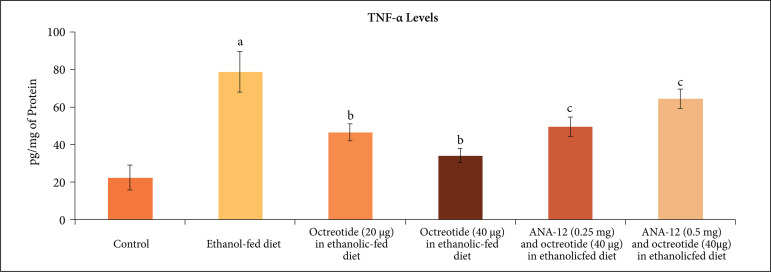
Effect of different treatments on TNF-α levels in the supernatant of nerve homogenate. **a** = p < 0.05 *vs*. control; **b** = p < 0.05 *vs*. ethanol fed diet; **c** = p < 0.05 *vs*. octreotide (40 µg·kg^–1^) in ethanolic-fed diet.

**Figure 9:**
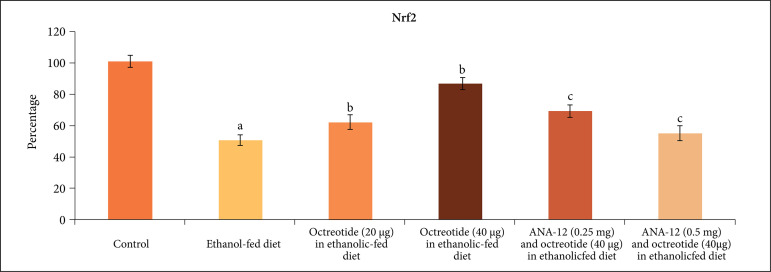
Effect of different treatments on Nrf2 levels in the supernatant of nerve homogenate. **a** = p < 0.05 *vs*. control; **b** = p < 0.05 *vs*. ethanol fed diet; **c** = p < 0.05 *vs*. octreotide (40 µg·kg^–1^) in ethanolic-fed diet.

In ethanol-fed animals, there were significant changes in the biochemical parameters along with the development of neuropathic pain. Specifically, there was a significant increase in the H_2_S levels (Fig. 4), CSE (Fig. 5) and CBS (Fig. 6) in the sciatic nerve homogenate. There was a significant increase in neuroinflammation, as assessed by an increase in TNF-α levels (Fig. 7). Moreover, the levels of BDNF (Fig. 8) and Nrf2 were also reduced significantly in the sciatic nerve homogenate in an ethanolic-fed diet (Fig. 9).

## Alterations in neuropathic pain and biochemical changes in response to treatment with octreotide and ANA-12

Treatment of ethanolic-fed animals with octreotide (20 and 40 µg·kg^–1^) for 28 days significantly attenuated mechanical allodynia (Fig. 1), cold allodynia (Fig. 2) and mechanical hyperalgesia (Fig. 3), suggesting the attenuation of neuropathic pain. Moreover, it also attenuated ethanol-induced biochemical changes including an increase in the H_2_S levels (Fig. 4), CSE (Fig. 5) and CBS (Fig. 6) in a dose-dependent manner. Moreover, it also decreased neuroinflammatory marker, TNF-α levels (Fig. 7) and increased the levels of BDNF (Fig. 8) and Nrf2 levels (Fig. 9). Co-administration of BDNF blocker (ANA-12, 0.25 and 0.5 mg·kg^–1^) attenuated the beneficial effects of octreotide and there was a significant increase in neuropathic pain in ANA-12 treated rats. ANA-12 also attenuated the effects of octreotide on the TNF-α and Nrf2. However, ANA-12 did not modulate the levels of H_2_S, CSE and CBS in octreotide-treated rats in a significant manner.

## Discussion

In the present study, administration of alcohol for 28 days led to significant development of neuropathic pain assessed in terms of mechanical allodynia (von Frey test), cold allodynia (acetone spray test) and mechanical hyperalgesia (pinprick test). Along with metabolic complications, chronic alcohol consumption is associated with pathological changes in the nervous system^2^, whose manifestation may be in the form of the development of neuropathic pain^1^^,^ 3. The present study results shows the development of neuropathic pain symptoms in rodents due to ethanol consumptions are in line with previous studies. Indeed, it has been shown that neuropathic pain begins after 28 days of ethanol administration^20^^,^ 26. Accordingly, the behavioral pain-related assessment was done after 28 days of alcohol consumption.

In this study, treatment with somatostatin analogue, i.e., octreotide, led to significant improvement in neuropathic pain manifestations. There have been studies showing that apart from endocrinological effects, octreotide produces a number of beneficial effects in different disease states, including ischemia-reperfusion injury^27^, depression^28^, dementia^29^. Administration of octreotide in the ventrolateral orbital cortex has been shown to produce antinociceptive effects in formalin-induced nociceptive behavior in rats^12^. Moreover, it has been shown to attenuate manifestations of diabetic neuropathy^13^. However, it is the first study showing the pain attenuating actions of octreotide in alcohol-associated neuropathy.

In the present study, administration of octreotide also normalized chronic alcohol consumption-induced biochemical alterations in the sciatic nerve. Octreotide normalized alcohol-induced decrease in the levels of H_2_S along with its biosynthetic enzymes, including CSE and CBS. Indeed, there was a decrease in the expression of H_2_S biosynthetic enzymes CSE and CBS in the sciatic nerve along with the decrease in the levels of H_2_S in the sciatic nerve in response to chronic alcohol consumption. There have been studies showing that a decrease in the H_2_S levels plays a critical role in the development of neuropathic pain^30^^,^ 31. Octreotide-induced normalization of H_2_S, CBS and CSE levels along with the improvement of neuropathic pain symptoms suggests that octreotide-mediated improvement in neuropathic pain manifestations may be secondary to an increase in H_2_S levels as a consequence of an increase in CBS and CSE expression.

Furthermore, octreotide treatment led to attenuation of alcohol-induced neuroinflammation assessed by a decrease in the TNF-α levels. Neuroinflammation plays a critical role in the development of neuropathic pain^32^^,^ 33 and there have been studies that an increase in H_2_S levels decreases neuroinflammation to attenuate neuropathic pain^34^^,^ 35. Therefore, it may be possible that an octreotide-mediated decrease in TNF-α levels may be secondary to an increase in the H_2_S levels. Moreover, there was a significant increase in the expression of BDNF and Nrf2 in the sciatic nerve in response to octreotide treatment in this study. BDNF belongs to the family of neurotrophic factors and its decreased levels may be important in the induction and maintenance of neuropathic pain^14^^,^ 36. Nrf2 is a transcriptional factor and is responsible for increasing the levels of endogenous antioxidants. The decrease in Nrf2 is also an important mechanism in inducing the development of neuropathic pain^37^. Accordingly, it may be possible that octreotide may increase the expression of BDNF and Nrf2 to confer protection to pain induction in response to chronic alcohol consumption. The role of BDNF in octreotide-mediated antinociceptive actions was supported by the results of the present study, showing that co-administration of BDNF blocker, ANA-12 abolished the neuropathic pain attenuating actions of octreotide. In other words, octreotide failed to exhibit its antinociceptive actions in the presence of ANA-12, BDNF receptor blocker. It suggests that octreotide-mediated antinociceptive actions are dependent on the increase in the expression of BDNF.

Co-administration of ANA-12 also attenuated the effects of octreotide on the TNF-α and Nrf2 levels and there was an increase in the levels of TNF-α and a decrease in the levels of Nrf2 in ANA-12 treated rats. It suggests that the changes in the TNF-α and Nrf2 levels are related to the actions of BDNF. There have been previous studies suggesting that BDNF decreases neuroinflammation and decreases the levels of TNF-α^38^, while it increases the levels of Nrf2^29^ to attenuate the neuropathic pain symptoms. However, ANA-12 did not modulate octreotide-mediated increase in H_2_S, CBS and CSE levels. It possibly suggests that the synthesis of H_2_S is not under the control of BDNF or both pathways are not related to each other. Alternatively, it is also possible that BDNF is a downstream mediator of H_2_S signaling and, thus, the BDNF blocker was unable to regulate the levels of H_2_S levels. Based on these, it may be concluded that octreotide attenuates the behavioral manifestations of alcoholic neuropathic pain, which may be due to an increase in H_2_S, CBS, CSE, BDNF, Nrf2 and a decrease in neuroinflammation. However, more studies are required to fully elucidate the precise relationship between BDNF and H_2_S signaling in octreotide-mediated beneficial effects in alcoholic neuropathic pain.
